# Accuracy Is Not Enough: Reasoning and Reference Reliability in Orthopaedic Large Language Model (LLM) Applications

**DOI:** 10.7759/cureus.100845

**Published:** 2026-01-05

**Authors:** Shashwat Singh, Pranav Chandrasekhar

**Affiliations:** 1 Trauma and Orthopaedics, The Queen Elizabeth Hospital King's Lynn NHS Foundation Trust, King's Lynn, GBR; 2 Medical School, The University of East Anglia, Norwich, GBR

**Keywords:** artificial intelligence in surgery, gpt-5, large language models (llms), llm, orthopaedic surgery

## Abstract

Background: Large language models (LLMs) now achieve performance comparable to senior postgraduate trainees on orthopaedic examinations and are increasingly trusted by clinicians to provide explanations for educational and decision-support purposes. However, correct answers do not necessarily indicate sound reasoning or reliable referencing. Current evaluations in this field emphasise accuracy alone, ignoring the quality and evidentiary reliability of the reasoning process.

Aim: This study aimed to systematically evaluate the relationship between answer accuracy, reasoning quality, and reference reliability in the latest generation of LLMs applied to a standardised postgraduate orthopaedic examination.

Methods: The 2024 Orthopaedic In-Training Examination (OITE; 203 questions) was administered to GPT-5 (OpenAI, San Francisco, CA, USA). The model was prompted to provide one answer, a brief rationale, and one supporting reference per question. Accuracy and percentile were recorded relative to official American Academy of Orthopaedic Surgeons (AAOS) data. A structured subsample of 88 responses (44 correct, 44 incorrect) underwent detailed validation of referencing and reasoning. GPT-5's reasoning was compared against official AAOS answer explanations for each question. Reasoning quality was scored using a three-point ordinal scale. References were categorised as fabricated, misrepresented, or accurate. Hallucination rates and reasoning scores were compared between correct and incorrect answers.

Results: GPT-5 achieved 78.3% accuracy (159/203), exceeding the OITE pass threshold (67%) and the mean postgraduate year-5 (PGY-5) resident score (73%), the highest accuracy reported, to our knowledge, among peer-reviewed studies to date. In the subset of 88 responses, hallucinations occurred in 33% overall, significantly higher in incorrect (50%) than in correct answers (15.9%; p=0.001). Reasoning among correct answers was consistently high (median 2.0, IQR 0.0), with 95.5% scoring maximum points, indicating reasoning entirely concordant with the reasoning provided by AAOS. Image-based questions showed lower accuracy (44.7%) compared with text-based questions (54%), though this difference was not statistically significant (p=0.52).

Conclusions: GPT-5 appears to exceed previously reported LLM performance on the OITE and achieved accuracy higher than published mean scores for senior trainees, but demonstrated poor reference reliability, with one in three answers citing fabricated or misrepresented evidence. Even correct answers frequently relied on flawed or unverifiable sources. Evaluation of LLMs in medical education should incorporate systematic reasoning and evidence validation, not accuracy alone.

## Introduction

Large language models (LLMs) are rapidly transforming medical education, examination preparation, and clinical decision support [1,2]. These systems, typified by OpenAI's GPT series (San Francisco, CA, USA), provide fluent explanations to complex clinical queries.

The American Orthopaedic In-Training Examination (OITE), administered annually by the American Academy of Orthopaedic Surgeons (AAOS), serves as a rigorous benchmark for evaluating trainee knowledge. Performance on the OITE correlates with success on the American Board of Orthopaedic Surgery Part I examination [3], making it an ideal tool for assessing LLM competence against established clinical standards.

Evaluation of earlier models of GPT showed iterative improvement in performance on the OITE. GPT-3.5 failed the 2020-2022 OITEs, achieving an average of 54.3% accuracy, equivalent to postgraduate year-1 (PGY-1) residents [4]. Multiple studies found GPT-4 passed the OITE, with one study quoting accuracy scores of 69.1%, increasing to 77.8% when textual descriptions of accompanying media were provided [5,6].

While accuracy metrics have improved across model generations, accuracy alone does not ensure reliable reasoning or evidence use. LLMs are known to fabricate references [7] or construct plausible but incorrect explanations [5,6]. Such tendencies risk misleading learners and clinicians who may assume fluent responses and high accuracy reflect reliable reasoning and referencing. As these models become increasingly accurate and so increasingly integrated into informal clinical decision-making and examination preparation, the disconnect between answer correctness and reasoning reliability poses significant educational and patient safety concerns.

No published study has yet assessed GPT-5 (the latest OpenAI model available to the public) on the OITE or systematically analysed the reliability of its reasoning and referencing. This study therefore benchmarked GPT-5's accuracy against human trainee performance, evaluated the reliability of its cited evidence, and assessed reasoning quality in correct responses.

## Materials and methods

Examination procedure

The 2024 OITE comprises 275 single-best-answer questions covering trauma, arthroplasty, paediatrics, spine, hand surgery, and basic science. The AAOS has made 203 of these questions available for public purchase online. We used this set of 203 questions to test GPT-5 in this study. No restricted or proprietary examination content was accessed.

GPT-5 was accessed between 29th and 30th August 2025 via the ChatGPT web interface on a desktop browser. Each question was entered in a new chat window to prevent cross-question memory effects. The temperature parameter was left at its default value of 1.0, ensuring typical stochastic behaviour of the publicly available interface.

A standardised prompt was applied uniformly to all 203 questions to ensure reproducibility: "You are an orthopaedic surgery resident taking the OITE. Please analyse this question and provide (1) your chosen answer, (2) brief reasoning for your choice, and (3) one relevant reference or guideline that supports your answer." The same prompt wording was used for both text- and image-based questions. Image-based questions were uploaded as high-resolution screenshots.

Percentile rank was recorded relative to AAOS national trainee data derived from performance on the same publicly released OITE question set for that examination year, as reported in the AAOS OITE 2024 technical report [8]. 

Subset validation

A subsample of 88 questions was selected for detailed validation. This included all 44 incorrect responses and 44 correct responses chosen at random for comparison using a pseudo-random number generator (RAND function, Microsoft Excel 2021 (Microsoft Corp., Redmond, WA, USA)). This approach ensured that every incorrect response was examined while maintaining a balanced comparison group of correct responses without selection bias. Each response was independently assessed for reasoning quality and reference reliability by a single reviewer. As the process involved one reviewer, no inter-rater reliability data are available.

Reference Validation

The first cited source for each answer was reviewed, and the claim made by GPT-5 was verified using PubMed, Google Scholar, and Google, with additional assistance from the hospital library to source textbooks when these were referenced. The reviewer was blinded to answer correctness during the verification process. If a reference could not be located on PubMed, on Google Scholar, or through librarian-assisted searches, it was deemed likely fabricated and documented as such. References were classified as misrepresented if they existed but either did not address the specific clinical topic cited or failed to directly support GPT-5's stated claim. If the reference was found and directly supported the claim made by GPT-5, it was classified as a supportive reference. For statistical analysis, fabricated and misrepresented references were collectively defined as hallucinations, while all others were considered accurate.

Reasoning Quality Assessment

The reasoning provided by GPT-5 for each question was compared with model reasoning accompanying each question provided by the AAOS as part of the online question bank. This comparison was made by a reviewer blinded to answer accuracy. Reasoning quality was assessed for correct answers only, as these were the "misleadingly correct" subset of answers that were of most interest to this study; reasoning accompanying incorrect answers was reviewed qualitatively but not formally scored. A three-point rubric was applied, aligned with OITE official discussion keys as follows: 0 (absent or irrelevant reasoning), 1 (partial alignment with expected clinical reasoning), and 2 (clear, structured reasoning concordant with official explanation).

Statistical analysis

Accuracy was expressed as a percentage correct. Hallucination rates were compared between correct and incorrect answers using Fisher's exact test. Reasoning scores were summarised as median with interquartile range (IQR). Accuracy for image-based versus text-only questions was compared using Fisher's exact test. Fisher's exact test computes exact p-values from the hypergeometric distribution and does not yield a conventional test statistic (χ², t, F); therefore, only exact two-tailed p-values are reported. All analyses were conducted using IBM SPSS Statistics for Windows, V. 29.0 (IBM Corp., Armonk, NY, USA), with statistical significance set at p<0.05.

Ethical and data-use statement

The OITE questions and associated material used in this study were derived from publicly available material accessible through the AAOS website. No proprietary or confidential question content was accessed. The study did not involve human participants, patient data, or institutional review board-regulated activity. All prompts, randomisation code, and raw GPT-5 outputs are archived by the authors and are available upon reasonable request to support reproducibility.

## Results

Overall accuracy

GPT-5 answered 159 of 203 questions correctly (78.3%), surpassing both the OITE pass threshold (67%) and the mean PGY-5 resident score (73%). This performance places GPT-5 in the 89th percentile of all test-takers, relative to national trainee data reported by the AAOS Technical Report 2024 [8].

Reference reliability

Across the subset of 88 responses, hallucinations (fabricated or misrepresented references) were observed in 29 cases (33%). Incorrect answers contained hallucinations in 50% (22/44) of cases compared with 15.9% (7/44) of correct answers, a statistically significant difference (Fisher's exact test, no conventional test statistic; p=0.001). This is described in Table 1. 

**Table 1:** Reference reliability by answer correctness

Answer type	Total	Hallucinations	Accurate citations	Hallucination rate (%)
Correct	44	7	37	15.9
Incorrect	44	22	22	50
Total	88	29	59	33

When stratified by reference type, hallucination rates varied substantially: clinical practice guidelines (41.7%), randomised controlled trials (25%), cohort studies (23.5%), review articles (23.1%), and textbooks (10%). No hallucinations were identified in citations to professional society websites or other grey literature (Table 2).

**Table 2:** Hallucination rates by reference type

Reference type	Total citations	Hallucinations	Hallucination rate (%)
Guidelines	12	5	41.7
Randomised controlled trials	8	2	25
Cohort studies	17	4	23.5
Reviews	26	6	23.1
Textbooks	20	2	10
Websites	3	0	0
Other	2	0	0
Total	88	29	33

Reasoning quality

Among the 44 correct answers, reasoning quality was uniformly high (median 2.0, IQR 0.0). Forty-two responses (95.5%) scored 2.0, one (2.3%) scored 1.0, and one (2.3%) scored 0.0. This ceiling effect suggests that when GPT-5 selects the correct answer, it typically generates reasoning that aligns closely with accepted clinical explanations.

Image-based versus text-based performance

Of the 88 questions in the validation subset, 38 were image-based and 50 were text-only. Image-based questions demonstrated lower accuracy (44.7%, 17/38) compared with text-based questions (54%, 27/50), though this difference did not reach statistical significance (Fisher's exact test, no conventional test statistic; p=0.52) (Table 3).

**Table 3:** Accuracy by question type

Question type	Total questions	Correct answers	Incorrect answers	Accuracy (%)
Image-based	38	17	21	44.7
Text-only	50	27	23	54
Total	88	44	44	50

## Discussion

This evaluation demonstrates that GPT-5 achieved expert-level accuracy on a comprehensive postgraduate orthopaedic examination, exceeding senior resident mean scores and so reflecting substantial progress since earlier GPT generations. GPT-5 provided high-quality reasoning for questions it answered correctly. However, GPT-5 exhibited major flaws in referencing, with one-third of its references being fabricated or misrepresented, and this weakness persisted in both correct and incorrect answers. For questions answered incorrectly, the accompanying reasoning was often incomplete or inconsistent with official explanations on qualitative review; however, these responses were not formally scored, as the primary focus of the study was on the potential for misleadingly correct answers.

High accuracy does not guarantee reliable reasoning

The findings reinforce that answer correctness alone is an inadequate indicator of LLM competence. This dissociation between outcome and process is particularly problematic in medical education, where learners are encouraged to understand not just what the answer is but why it is correct. Trainees who trust GPT-5's explanations uncritically may inadvertently internalise false claims or develop flawed clinical reasoning patterns based on non-existent literature [9].

Somewhat reassuringly, we found that when GPT-5's answer could be independently verified to be correct, the reasoning was usually of high quality. The uniformity of high reasoning scores (95.5% scoring maximum points) among correct answers reflects the model's ability to generate coherent explanations that align with standard clinical teaching. Importantly, in cases where access to easy and convenient verification of answer accuracy is not available (most use cases aside from answering questions from a question bank), knowledge that GPT-5's reasoning is high quality if the answer is accurate is of low utility.

Poor use of referencing and patterns of evidence hallucination

While GPT-5 achieved 78.3% accuracy, 15.9% of its correct answers relied on fabricated or misrepresented sources. Hallucinations clustered within clinical practice guidelines (41.7%), randomised controlled trials (25%), and cohort studies (23.5%), while textbooks showed relative reliability (10%). Clinical guidelines often iterate frequently, perhaps increasing the probability of citation errors due to the use of outdated guidance.

Our findings align with cross-disciplinary studies reporting 29-47% fabricated references in ChatGPT outputs [10,11]. The consistency of hallucination rates across medical domains suggests a fundamental architectural limitation rather than a domain-specific knowledge gap. Until LLMs incorporate robust fact-checking mechanisms or direct database access, citation unreliability will remain a significant barrier to clinical and educational deployment.

Image-based performance

The non-significant trend toward lower accuracy on image-based questions (44.7% versus 54%) should be interpreted with caution given the modest sample size and resulting limited statistical power. Unlike prior evaluations of earlier GPT models, which demonstrated a clear and statistically significant performance deficit on image-based OITE items [12], the present analysis did not identify a statistically significant difference in accuracy between image-based and text-only questions. This finding should not be interpreted as evidence of equivalence or proficiency in visual reasoning, but rather as the absence of demonstrable categorical underperformance in this limited subset. Larger, imaging-focused evaluations are required to confirm whether this observation reflects a true improvement in visual capabilities.

Implications for education and clinical use

LLMs offer potential as supplementary learning tools, providing answers rapidly, conveniently, and with expert-level accuracy. However, their tendency to fabricate or distort evidence and provide unreliable reasoning necessitates structured oversight. Educational use should be accompanied by explicit guidance that model-generated references must be independently verified before incorporation into clinical reasoning or scholarly work. Uncritical acceptance of LLM outputs risks propagating false citations and undermining evidence-based practice.

In clinical decision-support contexts, reference hallucination poses direct patient safety concerns. A clinician who relies on a fabricated guideline citation may make inappropriate management decisions with false confidence. Developers must therefore prioritise transparency, implement citation verification systems, and calibrate model outputs to express appropriate uncertainty [13]. Until these safeguards are in place, despite the high and increasing levels of accuracy that LLMs display, they should continue to be treated as preliminary information sources requiring expert validation rather than autonomous decision-support tools.

Implications for LLM evaluation

Current benchmarks for medical artificial intelligence (AI) often emphasise accuracy metrics derived from multiple-choice examinations. While these assessments provide useful performance bounds, they fail to capture the reasoning and evidence quality that underpin safe clinical practice. This study demonstrates that high accuracy can coexist with pervasive citation unreliability.

Future validation protocols should incorporate the systematic assessment of reasoning quality, citation accuracy, and explanatory coherence alongside traditional performance metrics. Multi-dimensional evaluation would provide a more honest characterisation of model capabilities and limitations, supporting more informed decisions about appropriate use cases and supervision requirements.

Limitations

This analysis is limited by its sample size (88 questions undergoing detailed validation) and by the use of a single reviewer for the assessment of reasoning and reference validity; although structured criteria were applied, no inter-rater reliability statistics could be calculated, and some subjectivity cannot be excluded.

While the OITE is a well-validated examination, it is US-centric and may not fully represent international orthopaedic curricula or clinical practice patterns. In addition, this study used the 203 OITE questions publicly released by the AAOS rather than the full 275-question examination; publicly released items may differ systematically from unreleased questions in difficulty, recency, content distribution, or proportion of image-based material, which may bias performance estimates. GPT-5 outputs are probabilistic and sensitive to sampling parameters; as only a single run was performed at the default temperature setting, the reported accuracy, reasoning scores, and hallucination rates represent a single performance snapshot and may vary across repeated runs.

The detailed validation subset was intentionally designed to include all incorrect responses with a randomly selected matched sample of correct answers; however, the modest subsample size limits formal power calculations and may reduce the generalisability of hallucination rates to the full dataset. Finally, the OITE's multiple-choice format constrains the assessment of open-ended reasoning or complex surgical decision pathways.

Broader multi-examination comparisons, multi-rater validation, repeated model runs, and inclusion of open-ended reasoning tasks would further strengthen confidence in these findings and support generalisability across medical domains.

## Conclusions

GPT-5 achieved accuracy comparable to or exceeding that of senior orthopaedic trainees on the 2024 OITE, demonstrating substantial progress from previously tested LLMs. However, one-third of its references were fabricated or misrepresented, and this unreliability persisted even in correct answers with otherwise sound reasoning. Accuracy alone therefore provides an incomplete and potentially misleading assessment of LLM competence in medical contexts.

As these models become increasingly integrated into medical education and informal clinical decision-making, evaluation frameworks must evolve beyond simple correctness metrics. Safe and effective deployment requires the systematic assessment of reasoning transparency, citation fidelity, and appropriate expression of uncertainty. Until such standards are established and met, clinicians and trainees should treat LLM-generated content as preliminary information requiring independent verification rather than authoritative guidance.
